# Monoacylglycerol lipase inhibitor JZL184 reduces neuroinflammatory response in APdE9 mice and in adult mouse glial cells

**DOI:** 10.1186/s12974-015-0305-9

**Published:** 2015-04-28

**Authors:** Rea Pihlaja, Jatta Takkinen, Olli Eskola, Jenni Vasara, Francisco R López-Picón, Merja Haaparanta-Solin, Juha O Rinne

**Affiliations:** MediCity/PET Preclinical Laboratory, Turku PET Centre, University of Turku, Tykistökatu 6 A, FI-20520 Turku, Finland; Turku PET Centre, Radiopharmaceutical Chemistry Laboratory, University of Turku, Turku, Finland; Turku PET Centre, Turku University Hospital, University of Turku, Turku, Finland

**Keywords:** Alzheimer’s disease, Neuroinflammation, Monoacylglycerol lipase

## Abstract

**Background:**

Recently, the role of monoacylglycerol lipase (MAGL) as the principal regulator of simultaneous prostaglandin synthesis and endocannabinoid receptor activation in the CNS was demonstrated. To expand upon previously published research in the field, we observed the effect of the MAGL inhibitor JZL184 during the early-stage proinflammatory response and formation of beta-amyloid (Aβ) in the Alzheimer’s disease mouse model APdE9. We also investigated its effects in proinflammatory agent - induced astrocytes and microglia isolated from adult mice.

**Findings:**

Transgenic APdE9 mice (5 months old) were treated with JZL184 (40 mg/kg) or vehicle every day for 1 month. *In vivo* binding of the neuroinflammation-related, microglia-specific translocator protein (TSPO) targeting radioligand [^18^ F]GE-180 decreased slightly but statistically non-significantly in multiple brain areas compared to vehicle-treated mice. JZL184 treatment induced a significant decrease in expression levels of inflammation-induced, Iba1-immunoreactive microglia in the hippocampus (*P* < 0.01) and temporal and parietal (*P* < 0.05) cortices. JZL184 also induced a marked decrease in total Aβ burden in the temporal (*P* < 0.001) and parietal (*P* < 0.01) cortices and, to some extent, in the hippocampus. Adult microglial and astrocyte cultures pre-treated with JZL184 and then exposed to the neuroinflammation-inducing agents lipopolysaccharide (LPS), interferon-gamma (IFN-γ), and Aβ_42_ had significantly reduced proinflammatory responses compared to cells without JZL184 treatment.

**Conclusions:**

JZL184 decreased the proinflammatory reactions of microglia and reduced the total Aβ burden and its precursors in the APdE9 mouse model. It also reduced the proinflammatory responses of microglia and astrocytes isolated from adult mice.

## Findings

During early-stage Alzheimer’s disease (AD), the neuroinflammatory responses of hyperactivated glial cells have a major impact on the development of AD pathology, including the formation of neurotoxic beta-amyloid (Aβ) [1,2]. Suppressing inflammatory signaling has had beneficial effects in animal models of AD [3]. On the other hand, molecular imaging of neuroinflammation may be employed for the early diagnosis of AD and to evaluate the patient’s response to treatment.

In the brain, the production of proinflammatory prostaglandins is mainly regulated by the activity of monoacylglycerol lipase (MAGL) [4]. MAGL hydrolyzes the anti-inflammatory and neuroprotective endocannabinoid 2-arachidonoylglycerol (2-AG) to arachidonic acid (AA), which is further metabolized to prostaglandins. Inhibition of MAGL by genetic manipulation or the MAGL inhibitor JZL184 reduces the levels of AA and its prostaglandin metabolites E2 and D2. Inhibition of MAGL also increases levels of 2-AG in the brain. Both of these mechanisms are associated with neuroprotection in a parkinsonian mouse model and alleviation of lipopolysaccharide (LPS)-induced neuroinflammation. Moreover, pharmacological [5] and genetic [6] inactivation of MAGL markedly suppresses Aβ load, reduces neuropathology, and improves cognitive function in the AD mouse models 5XFAD and PS1APP. To date, of the MAGL inhibitors, JZL184 has been the most thoroughly characterized *in vivo*; thus, this compound is widely used as a preclinical tool [7].

We extended the findings of previous studies by investigating the effect of JZL184 on the early-stage proinflammatory response and Aβ deposition in APPSwe/PSEN1dE9 (APdE9) [8] mice. The level of activated microglia in JZL184- and vehicle-treated mice was determined by positron emission tomography-computed tomography (PET/CT). Levels of gliosis and total Aβ were determined by immunohistochemistry. In addition, we studied the effect of JZL184 in glial cultures isolated from adult mice under conditions that mimic neuroinflammation.

In 3.5-month-old APdE9 mice, levels of proinflammatory mediators are correlated with soluble Aβ levels. By the age of 6 months, the proinflammatory responses of glial cells notably increased and the first Aβ deposits have formed [2]. For *in vivo* studies, we used 5-month-old female APdE9 mice. The studies were approved by the Animal Experiment Board of the Province of Southern Finland (licence number ESAVI-2010-04454/Ym-23). JZL184 (Cayman Chemical Company, Ann Arbor, MI, USA) formulation was prepared in an 18:1:1 solution of saline:emulphor:ethanol, as previously described [7]. Every day for 1 month, 40 mg/kg JZL184 was injected intraperitoneally into tg APdE9 mice (*n* = 7). Vehicle-treated tg APdE9 mice (*n* = 5) were used as controls.

**Figure 1:**
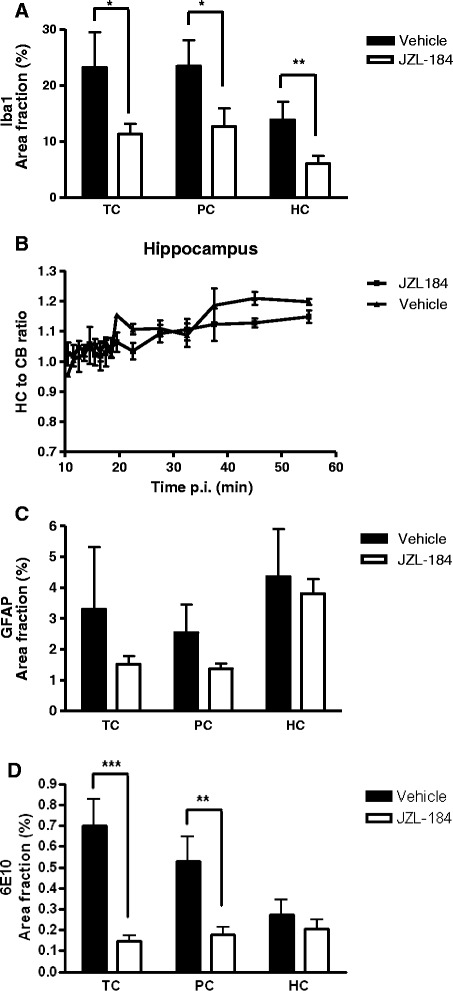
JZL184 treatment reduced the level of microgliosis and total Aβ in the AD mouse model. One month daily treatment with JZL184 (40 mg/kg, i.p.) reduced the immunoreactivity of **(A)** Iba1 in temporal (TC) (*P* < 0.05) and parietal (PC) (*P* < 0.05) cortices and hippocampus (HC) (*P* < 0.01) in 5-month-old tg APdE9 mice when compared to vehicle-treated mice. Also, **(B)** TSPO targeting radioligand [^18^ F]GE-180 decreased slightly but statistically non-significantly in multiple brain areas, for example, in the hippocampi. The level of **(C)** GFAP did not decrease significantly. The level of **(D)** 6E10 was reduced in TC (*P* < 0.001) and PC (*P* < 0.01) and slightly in HC.

Mice were anesthetized with 2.5% isoflurane, and 15 ± 2 MBq of radioligand [^18^ F]GE-180 [9] was injected intravenously. A 60-min dynamic PET/CT scan (Inveon multimodality PET/CT device; Siemens Medical Solutions, Malvern, PA, USA) was started immediately after injection (3D list mode; energy window of 350 to 650 keV; and 52 time frames: 30 × 10, 15 × 60, 4 × 300, and 2 × 600 s). Transmission scans were performed with CT for attenuation correction. Data were reconstructed with Fourier rebinning and a 2D-filtered back-projection reconstruction algorithm. Inveon Research Workplace Image Analysis software (Siemens Medical Solutions) was used to draw regions of interest (ROIs) over the whole brain, the cerebellum, and the entire cortex and separately over the frontal and parieto-temporal cortex, hippocampus, striatum, and thalamus. A 3D MRI template was used as an anatomical reference. Time-radioactivity curves were used to express the uptake of [^18^ F]GE-180 as % ID/g, and target-to-cerebellum ratios were calculated. In JZL184-treated mice, there was a very slight decreasing trend in translocator protein (TSPO) signal in multiple ROIs (for example, in the hippocampus, Figure 1B) compared to vehicle-treated mice. However, the difference was not statistically significant.

All JZL184 (*n* = 7) and vehicle (*n* = 5)-treated mice were anesthetized and transcardially perfused with heparinized saline. The cortex, hippocampus, and cerebellum were then isolated from the right hemisphere and snap frozen in liquid nitrogen. The left hemisphere was post-fixed with 4% paraformaldehyde solution, cryoprotected in 30% sucrose, snap frozen in liquid nitrogen, and stored at −70°C. Processing of the brain tissue and immunohistochemistry were performed as described previously [10], using the mouse beta-amyloid monoclonal antibody 6E10 (Covance Inc., Princeton, NJ, USA) and a polyclonal rabbit anti-glial fibrillary acidic protein (GFAP) antibody (Dako Cytomation, Glostrup, Denmark) that recognizes activated astrocytes or a rabbit polyclonal anti-Iba1 antibody (WAKO Pure Chemical Industries, Ltd., Osaka, Japan). The 6E10 antibody recognizes amino acid residues 1–16 in various abnormally processed isoforms of intra- and extracellular human Aβ [11-15], as well as amyloid precursors. Alexa Fluor 568-conjugated goat anti-rabbit (Gibco, Carlsbad, CA, USA) or donkey anti-mouse IgG (Dylight 488, Abcam, Cambridge, UK) were used as secondary antibodies. Images were acquired with the Leica DMR fluorescence microscope (Leica Microsystems GmbH, Wetzlar, Germany) and ISCapture software 2.6 (Fuzhou Xintu Photonics Co, Ltd., Fujian, China); settings were kept constant for quantitation. Six or seven sections per mouse with five to seven areas per section were imaged throughout the hippocampal formation. The areas examined were the temporal and parietal cortices and the hippocampus (including CA1, CA2, and DG). The quantification was performed using per area (%) method (Image J 1.43 U, Wayne Rasband, NIH, MA, USA).

**Figure 2:**
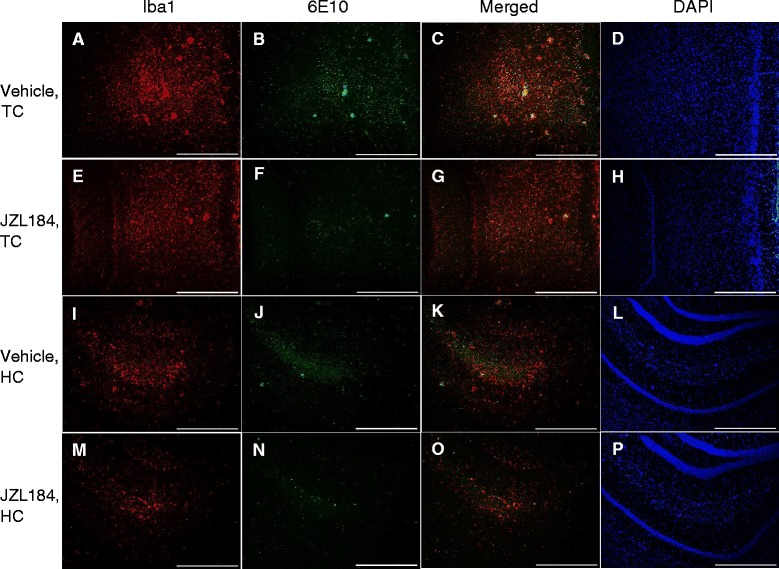
The level of microgliosis and total Aβ burden in JZL184 and vehicle-treated tg APdE9 mice. Similar to Figure 1, JZL184 treatment reduced Iba1 immunoreactivity in **(A, E)** temporal cortex (TC) and **(I, M)** hippocampus (HC) and 6E10 immunoreactivity in **(B, F)** TC and slightly in **(J, N)** HC of tg APdE9 mice when compared to mice treated with vehicle only. Merged images from TC **(C, G)** and from HC **(K, O)**. DAPI-stained nuclei from the responsive areas in **D**, **H**, **L** and **P**. Scale bars indicate 500 μM.

Levels of Iba1 expression were decreased in JZL184-treated mice in the temporal and parietal cortices (by 49% and 46%, respectively; *P* < 0.05) and in the hippocampus (by 57%, *P* < 0.01) compared to vehicle-treated mice (Figures 1A and 2A, E, I, M). Similarly, expression levels of GFAP decreased slightly but not significantly in all of the regions examined (Figure 1C). Levels of 6E10 immunoreactivity were reduced significantly in the temporal (by 79%, *P* < 0.001) and parietal (by 67%, *P* < 0.01) cortices and, to some extent, in the hippocampus compared to vehicle-treated mice (Figures 1D and 2B, F, J, N).

A human Aβ_42_ enzyme-linked immunosorbent assay (ELISA) was performed for the hippocampi and cortices isolated from the APdE9 mice, in accordance with the protocol for the Aβ42 Human ELISA Kit (Biosource International/Invitrogen, Carlsbad, CA, USA). Expression levels of Aβ_42_ in the hippocampus and cortex in ELISA did not differ between JZL184- and vehicle-treated tg APdE9 mice (data not shown). In contrast to 6E10 antibody, Aβ_42_ antibody in ELISA recognizes only C-terminus of the 1–42 Aβ sequence.

For the cell culture experiments, adult microglia and astrocytes were cultured according to the procedures outlined in [16] and [17], respectively. Three days before exposure, the cells were seeded (2 × 10^4^ cells/well) into 48-well plates in the absence of recombinant murine granulocyte-macrophage colony-stimulating factor (rmGM-CSF) and G5 supplement.

Homogenous Aβ_42_ (American Peptide Company, Sunnyvale, CA, USA) oligomers were prepared according to protocol by Dahlgren et al. [18]. For cell culture assays, the wells containing adult microglia and astrocytes were rinsed with medium w/o serum, incubated for 30 min in the presence or absence of 1 μM JZL184, and then exposed to 10 μM Aβ_42_ or a combination of 1 μM LPS and 100 ng/ml interferon-gamma (IFN-γ) for 24 h. The medium was collected for nitric oxide (NO) measurement, and the cells were fixed with 3.7% formaldehyde, rinsed, and stored in phosphate-buffered saline (PBS).

**Figure 3:**
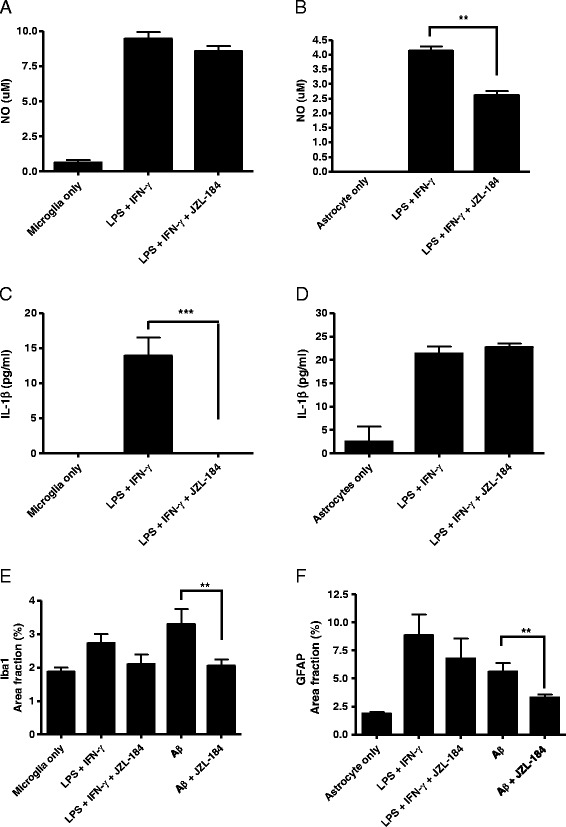
JZL184 decreased secretion of NO and IL-1β from adult glial cells exposed to proinflammatory agents. Astrocytes and microglia were pre-incubated with MAGL inhibitor JZL184 for 30 min before inducing proinflammatory responses by simultaneous exposure of 1 μM LPS and 100 ng/ml IFN-γ. After 24 h of incubation, secretion of NO was reduced **(A)** slightly in microglia and **(B)** significantly (*P* < 0.01) in astrocytes treated with JZL184 compared to exposed cells w/o JZL184 treatment. JZL184 induced **(C)** a marked reduction in IL-1β secretion from microglia (*P* < 0.001) but **(D)** did not have an effect on astrocytes compared to cells w/o JZL184 treatment. Pre-treatment with JZL184 decreased **(E)** Iba1 and **(F)** GFAP expression slightly in microglia and astrocytes induced with LPS and IFN-γ and significantly in microglia and astrocytes induced with Aβ_42_ (*P* < 0.01).

The concentration of NO in the cell medium was measured by Griess reaction according to the manufacturer’s instructions (Sigma-Aldrich). Fifty microliters of Griess reagent was then added to each well. The plate was shaken for 10 min, and the absorbance was measured at 560 nm (Wallac Victor2 V 1420; Wallac Oy, Finland). Levels of secreted IL-1β were determined by ELISA according to the manufacturer’s instructions (Biosource International/Invitrogen). Simultaneous exposure to LPS and IFN-γ was associated with a marked increase in the secretion of NO and IL-1β in both microglia and astrocytes, compared to unexposed cells (Figure 3A, B, C, D). A 30 min pre-treatment with JZL184 decreased NO secretion slightly in microglia (Figure 3A) and significantly in astrocytes (*P* < 0.01, Figure 3B) compared to cells w/o JZL184 treatment. JZL184 pre-treatment abolished the secretion of IL-1β in microglia (*P* < 0.001, Figure 3C) but not in astrocytes (Figure 3D) compared to cells w/o JZL184 treatment.

For immunocytochemistry, fixed cells were blocked with 10% normal goat serum (NGS) (Chemicon) in PBS with Tween-20 (PBST), incubated overnight with either anti-GFAP (in 5% NGS in PBST) or anti-Iba1-antibodies, and washed in PBST 3× for 5 min. The secondary antibodies, Alexa Fluor 568 goat anti-rabbit antibody (Gibco) or donkey anti-mouse IgG (Dylight 488, Abcam), were added to the cells, incubated for 2 h RT, washed, embedded in mounting medium, and imaged with a Zeiss Axiovert 200 M microscope, a Zeiss Axiovert MRc camera, and Axiovision 4.8 software (Zeiss International, Oberkochen, Germany). Four microscopic fields and four to five wells per cell type were analyzed for the percentage of anti-GFAP or anti-Iba immunostained cells, quantified using per area (%) method (Image J 1.43 U).

Aβ_42_ or simultaneous LPS and IFN-γ exposure increased Iba1 and GFAP expression. Pre-treatment with JZL184 decreased Iba1 expression significantly in microglia induced with Aβ_42_ (*P* < 0.01) and slightly when induced with LPS and IFN-γ (Figure 3E). Similarly, JZL184 pre-treatment significantly reduced GFAP expression in astrocytes induced with Aβ_42_ (*P* < 0.01) and slightly when induced with LPS and IFN-γ (Figure 3F).

The data were analyzed with *t*-test, one-way ANOVA with Tukey’s multiple comparison test, or Mann–Whitney *U*-test with GraphPad Prism v4.0. Significance was assumed if the *P* value was <0.05.

## Abbreviations

2-AG: 2-arachidonoylglycerol
AA: arachidonic acid
AD: Alzheimer’s disease
Aβ: beta-amyloid
CA: cornu ammonis
CT: computed tomography
DG: dentate gyrus
DMSO: dimethyl sulphoxide
ELISA: enzyme-linked immunosorbent assay
GFAP: glial fibrillary acidic protein
Iba1: ionized calcium binding adapter molecule-1
IFN-γ: interferon-gamma
LPS: lipopolysaccharide
MAGL: monoacylglycerol lipase
NaNO_2_: sodium nitrite
NGS: normal goat serum
NO: nitric oxide
PBS: phosphate-buffered saline
PBST: phosphate-buffered saline with Tween-20
PET/CT: positron emission tomography-computed tomography
ROI: region of interest
tg: transgenic
TSPO: translocator protein
